# Glances at the Insane in Other Lands

**Published:** 1889-03-02

**Authors:** 


### Glances at the Insane in Other Lands.

By a Medical Superintendent.

(*Continued from p. 263.*)

### V.—THE NEW BRUNSWICK ASYLUM, ST. JOHN.

Previous to the year 1848, the insane portion of the colony or province of New Brunswick was but indifferently cared for. There was certainly an institution of some kind in St. John for lunatics, nevertheless many patients must have been treated, or rather not treated, at home who would under proper conditions have recovered; others were sent far from their native province, and shut up amongst strangers in one of the hospitals for the insane in the United States, where they would have to associate with a foreign people, and where they would seldom be cheered by visits from their own friends. The following anecdote is related by Dr. Steeves, the medical superintendent of the New Brunswick Asylum: "Many years ago, before there was an insane asylum on Lancaster Heights, a gentleman resident in the city of Saint John, whose son became alienated in mind, seeing that there was no suitable place for his treatment at home, concluded to place him under care in an institution in Massachusetts. Father and son took passage in a steamer, in due time arrived at Boston, put up at an hotel, and on the following morning set out for the asylum, which they soon reached, entered, were shown into the waiting-room, and sat waiting the entrance of the doctor. The son was not in ignorance of the intention of his parent, nor was he agreed to the propriety of the proposed step; faulty though his mind was certified to be, he had mentally laid his plans to defeat his governor. Immediately upon the entrance of the doctor, the son arose, stepped forward and addressed him, saying, "I have brought this old gentleman here to place him under your charge for treatment. The father interposed, showing not a little discomfiture and excitement, urging that it was the son who was crazy; but the son commanded enough composure to at once inform the doctor that the old man believed everyone was crazy but himself, and suggested withdrawing to another room, and sending for attendants immediately, as his father, when opposed, was very violent and dangerous. The doctor was hoodwinked, the attendants were called, who seized the protesting, frenzied old man and placed him in a ward." It is a pity to spoil a good story; but one would like to know how the son got hold of the certificates, and how he managed to alter the statement of the age of the patient.

The Asylum for the province was opened on December 12th, 1848, and we learn from Table 29 of the Appendix attached to the report under notice, that ninety-two patients were resident on the last day of December, 1849. In 1887, the number had risen to 455, and the cry there as here is, "still they come." The percentage of recoveries would seem to vary greatly. In some years it was as high as 57, and in others as low as 29, calculated on the admissions. During last year 125 patients were admitted, 37 recovered, and 41 died. Of the admissions 104 were natives of the Dominion of Canada, 14 were Irish, 1 was Scotch, 5 were English, and 1 was a United States man. The total number under treatment was 560. Of these 525 were wholly supported by the Province, 7 were partially so, and 27 were private patients. The average cost per head per annum was ninety dollars and a-half, representing about nineteen pounds of our money—a rate by no means high for an asylum of that size.

Table 27 gives what we do not remember to have met with before in any asylum report, namely, the colour of the hair of those admitted. Taking the returns of the last twelve years we see that there were 1,493 admissions, of whom 422 had dark hair, 304 had brown hair, 212 had dark brown, 127 had grey, 60 had brown-grey, 86 black and grey, 112 had "dark" hair, 113 "light," 33 had auburn, and only 24 had red. The table is one the use of which we fail to see; but the man who is fond of drawing inferences may infer from the table either that red-haired people rarely become insane or that red hair is not common in New Brunswick.

The fabric of the asylum has undergone considerable modifications and additions lately. Twelve years since it was large enough to accommodate a little over 200 patients, although it then contained 258. To remedy this overcrowding, resort was had to the doubtful expedient of fitting up the basement as a dormitory for forty beds. In 1878 an addition for sixty beds was made on the male side of the asylum, and in 1881 a similar addition was made on the female side.

Before this time the wards were innocent of decoration; now every one is embellished, and a complete transformation has been effected. In 1884 the estate was enlarged by the purchase of 250 acres of land, and on this a building was erected capable of containing a hundred patients. The following paragraph is worthy of a wider circulation than it would be likely to get in an asylum report, and we therefore quote it verbatim: "The friends of insane patients are generally very willing to accept advice, more or less authoritative, which appears to justify them in attempting to carry out treatment at home. Against the affection which prompts this desire on the part of friends I have no condemnation or unkind word to utter, but it does appear to me that those whose intellectual natures mostly dominate over their affective, and who occupy the place of advisers, might well afford, when issues of momentous importance are at stake, to set forth in a candid, fair, and ingenuous light the facts and probabilities in these cases. It has been claimed, leaving out few exceptional cases, that insanity can be more successfully treated in an insane hospital than at home, and rarely has this claim been challenged so far as I know."

There is a curious clause in the provincial law relating to the insane. It runs: "Any person so disordered in his senses as to be dangerous when at large, may, on evidence of the fact, be apprehended and conveyed to the Provincial Lunatic Asylum." Dr. Steeves is of opinion that the sooner the word "dangerous" is expunged the better it will be for the insane; and in this opinion we fully concur. How such a word ever came to be inserted is a mystery, and it does not say much for the psychological knowledge of the framers of the Act.

(*To be continued.*)

